# High-performance ultraviolet photodetectors based on solution-grown ZnS nanobelts sandwiched between graphene layers

**DOI:** 10.1038/srep12345

**Published:** 2015-07-22

**Authors:** Yeonho Kim, Sang Jin Kim, Sung-Pyo Cho, Byung Hee Hong, Du-Jeon Jang

**Affiliations:** 1 Department of Chemistry, Seoul National University, Seoul 151-747, Korea; 2 National Center for Inter-University Research Facilities, Seoul National University, Seoul 151-747, Korea

## Abstract

Ultraviolet (UV) light photodetectors constructed from solely inorganic semiconductors still remain unsatisfactory because of their low electrical performances. To overcome this limitation, the hybridization is one of the key approaches that have been recently adopted to enhance the photocurrent. High-performance UV photodetectors showing stable on-off switching and excellent spectral selectivity have been fabricated based on the hybrid structure of solution-grown ZnS nanobelts and CVD-grown graphene. Sandwiched structures and multilayer stacking strategies have been applied to expand effective junction between graphene and photoactive ZnS nanobelts. A multiply sandwich-structured photodetector of graphene/ZnS has shown a photocurrent of 0.115 mA under illumination of 1.2 mWcm^−2^ in air at a bias of 1.0 V, which is higher 10^7^ times than literature values. The multiple-sandwich structure of UV-light sensors with graphene having high conductivity, flexibility, and impermeability is suggested to be beneficial for the facile fabrication of UV photodetectors with extremely efficient performances.

Photodetectors are a type of electronic devices for sensing light, and they have been found to have broad applications including flame monitoring, missile-plume detection, and space communication^1^,^2^,^3^,^4^. Wide bandgap semiconductor nanostructures such as ZnS, ZnO, and GaN as well as graphene quantum dots have been utilized as photoactive materials of photodetectors^3^,^4^,^5^,^6^,^7^,^8^,^9^,^10^. In particular, ZnS is a well-known semiconductor having a direct band gap of 3.67 eV, and it has been studied as functional parts in visible-blind ultraviolet (UV) photodetectors owing to its excellent optoelectronic properties^11^,^12^. Among ZnS nanomaterials, one-dimensional (1-D) nanostructures of ZnS have been known to be highly attractive building blocks for high-performance photodetectors^5^,^11^. The large surface-to-volume ratios and the deep-level surface trap states of 1-D nanostructures greatly prolong the lifetime of photocarriers and the reduced dimensionality of the active area in low-dimensional devices shortens the carrier transit time^13^,^14^. However, UV-light photodetectors constructed with 1-D ZnS nanostructures still display a weak photocurrent and unsatisfactory stability^15^,^16^,^17^,^18^. In order to overcome these limitations, hybridized structures have been attracted great attention owing to their superior optoelectronic properties compared to corresponding individual materials^19^,^20^,^21^,^22^,^23^. Graphene is a single atomic layer consisting of carbon atoms arranged in a hexagonal honeycomb lattice, and it has been reported as one of the fascinating two-dimensional materials due to its outstanding physical properties including high mechanical flexibility^24^,^25^, electrical conductivity^26^,^27^,^28^,^29^, and transparency^30^. In addition, graphene has an excellent stability under ambient conditions due to its outstanding impermeability^31^,^32^,^33^. The incorporation of transparent and conductive graphene into photoactive semiconductors can be considered to provide synergistic effects in light absorption and carrier transportation. In recent studies, the hybridization of graphene and semiconductors has demonstrated enhanced performances for UV-light photodetection, although their heterostructures still suffer from the small area of the effective-junction region contributing to the photocurrent^34^,^35^,^36^.

In this work, we present that high-performance UV photodetectors showing stable on-off switching and spectral selectivity have been fabricated via a facile process based on the hybrid structure of solution-grown ZnS nanobelts and CVD-grown graphene. Sandwich structures and multi-layer stacking strategies have been applied to the photodetectors for the increment of the effective-junction region between graphene and ZnS. Three kinds of UV photodetectors, ZnS spin-coated on the surface of double-layer graphene (D-G/ZnS), ZnS sandwiched between two graphene layers (S-G/ZnS), and multiply sandwiched graphene and ZnS (MS-G/ZnS), have been demonstrated in ambient conditions at a low bias voltage. The photo-response behavior of a photodetector has been found to depend considerably on the stacking sequence of graphene and ZnS, as well as on the number of layers. An optimized photodetector based on S-G/ZnS shows a high photocurrent of 37 μA, which is higher 10^6^ times than the reported values of graphene-free UV photodetectors based on ZnS nanobelts^16^ and ZnS-ZnO nanowires^11^.

## Results

### Schematic fabrication of graphene/ZnS photodetectors

**Figure 1:**
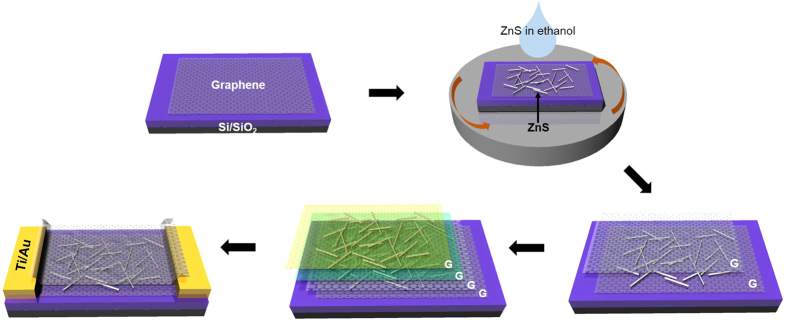
Schematic for the fabrication of a G/ZnS device. (**a**) graphene transfer, (**b**) ZnS spin coating, (**c**) graphene stacking, (**d**) repeated b and c steps for multiple coating and stacking, and (**e**) device fabrication with metal electrodes.

Figure 1 shows the fabrication schematics of a graphene and ZnS (G/ZnS) sandwich-structured device. Following graphene transfer on a Si/SiO_2_ wafer (Fig. 1a), a ZnS-dispersed ethanol solution was spin-coated on the graphene to fabricate the hybrid structure of G/ZnS (Fig. 1b). While Fig. 1c shows the stacking of graphene on the G/ZnS surface, Fig. 1d designates repeated steps to stack G/ZnS layers multiply. An additional graphene layer was well-stacked on a G/ZnS surface owing to the highly mechanical and flexible properties of graphene. The multiply stacked structure of G/ZnS is suggested to increase the effective-junction region of ZnS nanobelts and graphene as well as the absorbance of UV light. In addition, the overlying layer of impermeable graphene protects ZnS nanobelts from reactive environments. Fig. 1e shows the device fabrication of a UV-sensitive photodetector based on the prepared G/ZnS hybrid structure and metal electrodes (Ti/Au).

### Fabrication of ZnS nanobelts

**Figure 2:**
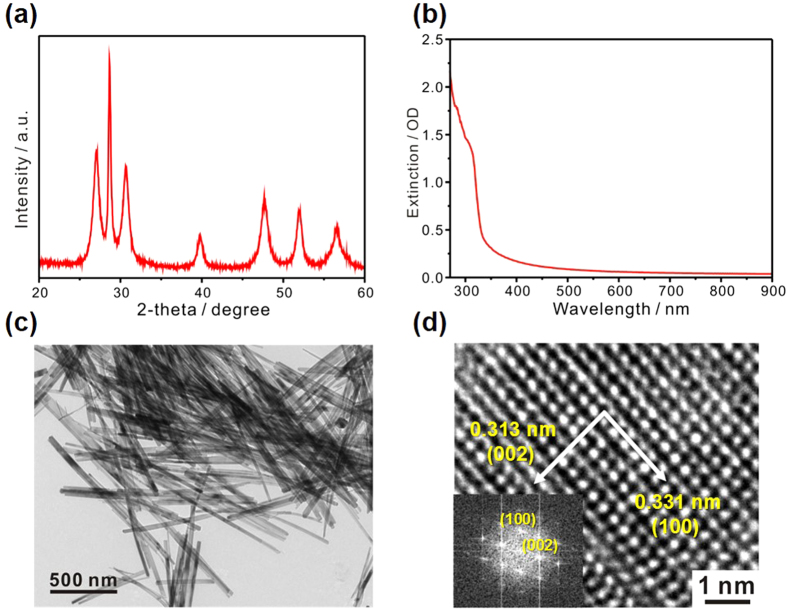
Characterization of ZnS nanobelts. (**a**) XRD pattern, (**b**) extinction spectrum (in ethanol), (**c**) TEM image, and (**d**) HRTEM image with an inserted FFT pattern of solution-grown ZnS nanobelts.

Figure 2a shows that the X-ray diffraction (XRD) pattern of solution-grown ZnS nanobelts can be indexed to the standard pattern of the reference wurtzite ZnS without having obvious impurity peaks (JCPDS Card No. 36-1450). However, the diffraction peak at 2-theta of 28.5°, indexed as the (002) planes, surpasses diffraction peaks arising from any other planes, supporting that ZnS nanobelts have grown preferentially to the direction of [001]. The extinction spectrum of Fig. 2b indicates characteristic absorption at 310 nm, which is one of the typical intrinsic optical features of ZnS nanomaterials giving rise to practical applications for photodetectors having spectral selectivity in the ultraviolet region. The transmission electron microscopy (TEM) image of Fig. 2c and the high resolution TEM (HRTEM) image of Fig. 2d display that as-prepared ZnS nanobelts have well-defined geometry with clear lattice fringes. The marked spacings of 0.313 nm and 0.331 nm in Fig. 2d agree well with the expected separations of the (002) and the (100) planes in the reference wurtzite ZnS, respectively. The inserted Fast Fourier Transform (FFT) pattern of the HRTEM image indicates the single-crystalline characteristics of our ZnS nanobelts with preferred growth along the [001] orientation.

### Characterization of graphene/ZnS hybrid structures

**Figure 3:**
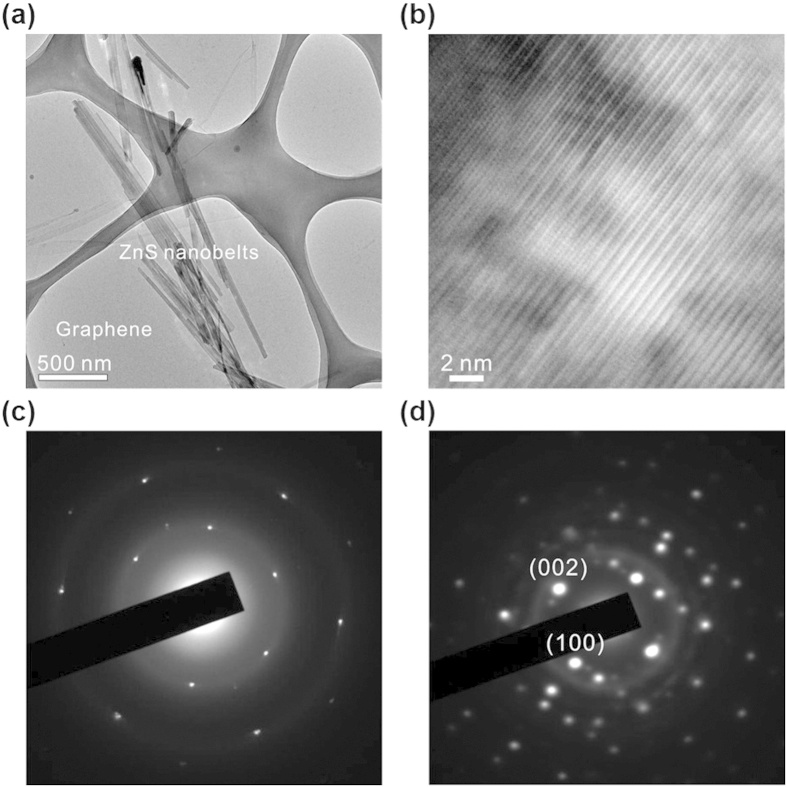
Characterization of graphene and S-G/ZnS. (**a**) TEM and (**b**) HRTEM images of S-G/ZnS and SAED patterns of (**c**) graphene and (**d**) S-G/ZnS.

The TEM image of Fig. 3a shows that ZnS nanobelts having a mean length of 2 μm, an average width of 80 nm, and a typical thickness of 16 nm lie closely each other on a graphene layer; ZnS nanobelts are fractured and aggregated during the sonication process of ZnS nanobelts-dispersed ethanol before spin coating onto the graphene-transferred substrate. Supplementary Fig. 1 shows the digital image of an as-fabricated device with a channel length of 80 μm. The HRTEM image of Fig. 3b indicates that a highly single-crystalline ZnS nanobelt covers a graphene sheet without having any stacking faults at the atomic level. The Raman spectrum of S-G/ZnS in Supplementary Fig. 2a shows two noticeable peaks of the G-band (at 1580 cm^−1^) and the 2D-band (at 2692 cm^−1^)^37^ of graphene with weak peaks of ZnS (at 250 and 350 cm^−1^)^38^. The mapping profiles in Supplementary Figs 2b–d indicate that graphene sheets are well covered with the photo-responsive nanobelts of ZnS. The selected-area electron diffraction (SAED) pattern of Fig. 3c taken in a graphene-only region displays the presence of the hexagonal symmetry of graphene indeed. The SAED pattern taken in a G/ZnS hybrid region (Fig. 3d) shows that ZnS is single-crystalline and well-aligned along the [001] direction of the wurtzite phase. The diffraction intensity of graphene is too weak compared to that of ZnS, so that it is rather difficult to observe the electron diffraction patterns of ZnS and graphene simultaneously in our experimental conditions.

### Characterization and evaluation of photodetectors

**Figure 4:**
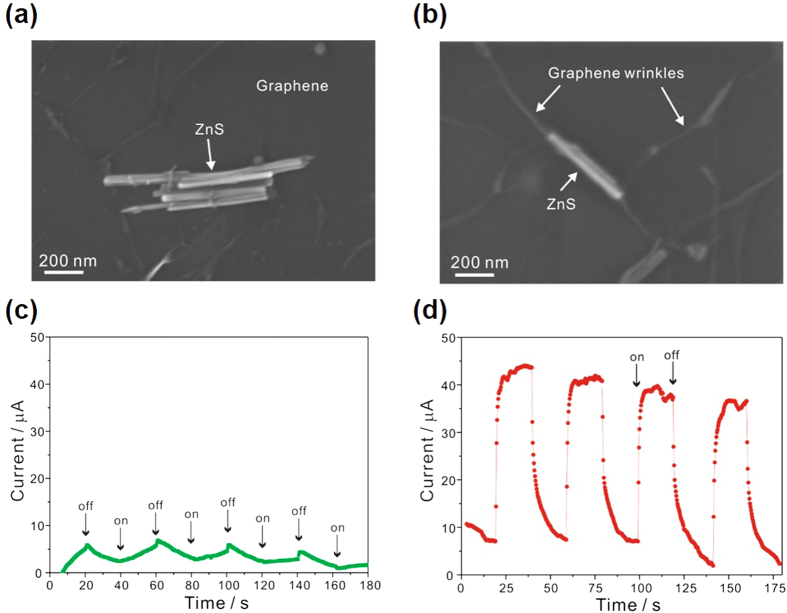
Characterization and evaluation of photodetectors. SEM images of (**a**) D-G/ZnS and (**b**) S-G/ZnS and response behaviors of photodetectors measured in air at a bias of 1.0 V based on (**c**) D-G/ZnS and (**d**) S-G/ZnS under 300 nm light illumination.

Figure 4a,b indicate the scanning electron microscopy (SEM) images of D-G/ZnS and S-G/ZnS, respectively; Fig. 4b shows the existence of large graphene wrinkles along ZnS nanobelts due to the flexible nature of graphene lying on a G/ZnS-coated substrate. Atomic force microscopy (AFM) images and their corresponding height profiles in Supplementary Fig. 3 also display that large graphene wrinkles are present abundantly in S-G/ZnS composites. These results imply that intermolecular interactions between graphene and ZnS has led graphene sheets to cover ZnS nanobelts tightly to form tent-like structures, as indicated in Supplementary Fig. 3b, which expand the effective-junction region of G/ZnS hybrid structures extensively. The current-voltage characteristic curves of graphene and a G/ZnS hybrid structure (Supplementary Fig. 4) are very similar to each other to have almost the same Dirac voltage, implying that the coating process of ZnS hardly brings any doping effect in graphene. Figure 4c,d present the time-dependent photoresponse behaviors of our devices measured with turning 300 nm light of 1.2 mW cm^−2^ on and off periodically in ambient conditions. The net photocurrent can be obtained by subtracting the dark current from the light current. For a S-G/ZnS photodetector (Fig. 4d), a high net photocurrent of 37 μA was recorded at a low bias of 1.0 V, while a low photocurrent of 4 μA was measured for a D-G/ZnS device (Fig. 4c). These results imply that compared with the D-G/ZnS structure, the sandwich structure of S-G/ZnS is of great advantage to charge transfer from ZnS to graphene due to the increase of the effective-junction region between photoactive ZnS nanobelts and graphene channels. The detector current photoresponsivity (*R*_*λ*_), defined as the photocurrent generated per unit power of the incident light on the active area of the device, and the external quantum efficiency (EQE), defined as the number of electrons detected per incident photon, are crucial parameters for photodetectors^39^. R_λ_ and EQE can be expressed as *I*_*λ*_*/(P*_*λ*_*S)* and *hcR*_*λ*_*/(eλ)*, respectively, where *I*_*λ*_ is the difference between the photocurrent and the dark current, *P*_*λ*_ is the light power, *S* is the active area of the device, *h* is the Planck’s constant, c is the velocity of light, *λ* is the excitation wavelength, and e is the electronic charge. Our as-fabricated device has a channel length of 80 μm and a channel width of 20 μm. Therefore, the active area of the device is 1.6 × 10^−5^ cm^2^. According to our experimental results, *R*_*λ*_ and EQE for a S-G/ZnS photodetector have been calculated as 1.9 × 10^3^ AW^−1^ and 8.0 × 10^5^%, respectively, under an applied bias voltage of 1 V with 300 nm illumination of 1.2 mWcm^−2^. The response time is another important parameter of a photodetector. The rise and decay times (defined as times required for the peak photocurrent to increase from 10% to 90% and to drop from 90% to 10%, respectively) of a S-G/ZnS photodetector have been measured as 2.8 and 7.5 s, respectively. The multiple stacking of graphene and ZnS (MS-G/ZnS) has facilitated the photocurrent enhancement up to 115 μA (Supplementary Fig. 5). In order to confirm the visible blindness of a photodetector, we have also measured the response behaviors of a S-G/ZnS photodetector under visible-light illumination of 1.2 mWcm^−2^ at three different wavelengths of 400, 500, and 600 nm (Supplementary Fig. 6); no obvious response has been observed under visible-light illumination. To confirm the repeatability and stability of our photodevice, we have also measured the photocurrent of a S-G/ZnS photodetector kept under ambient conditions for six months. Supplementary Fig. 7 indicates that the photocurrent generation of a S-G/ZnS photodetector has remained almost invariant for six months, demonstrating the outstanding repeatability and stability of our photodevice.

### Working mechanism of photodetectors

**Figure 5:**
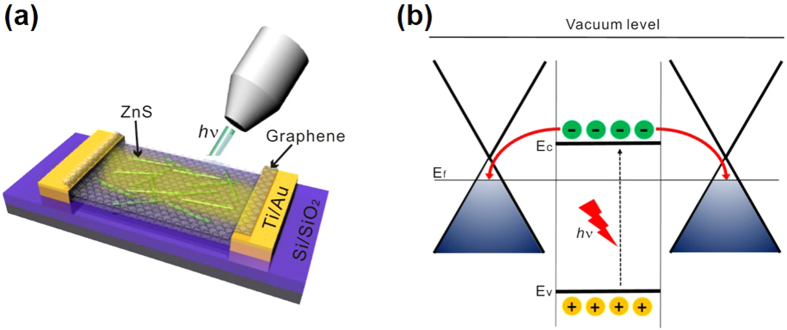
Diagram and working mechanism of a photodetector. (**a**) Schematic diagram of a S-G/ZnS photodetector under light illumination and (**b**) energy level diagram of S-G/ZnS heterojunctions showing the charge-transfer process under UV-light illumination.

Figure 5a,b show the schematic diagram and the charge-transfer mechanism of a S-G/ZnS photodetector under light illumination, respectively. Upon absorption of light over the band-gap energy of 3.82 eV (324 nm), electron-hole pairs are generated in photoactive ZnS nanobelts. Because the conduction-band level of ZnS is higher than the Fermi level of graphene, photoexcited electrons in the conduction band of ZnS nanobelts spontaneously undergo a charge-transfer process to graphene channels. In addition, the high charge-carrier mobility of graphene reduces the recombination process of electron-hole pairs drastically, increasing the generation of the photocurrent extremely^40^. This is the UV-selective photo-detection mechanism of S-G/ZnS nanocomposites that makes our sandwich-structured photodevices show high performances at a low bias in ambient conditions for UV-light detection.

## Discussion

In summary, we have developed high-performance UV photodetectors based on solution-grown ZnS nanobelts sandwiched between CVD-grown graphene sheets. The formation of tent-like graphene structures in S-G/ZnS has made ZnS nanobelts completely enclosed in graphene sheets with maintaining their original shapes and properties. The increment of the effective-junction region between graphene and photoactive ZnS nanobelts by the sandwiched structure has been attributed to bring about a considerably enhanced photocurrent under UV-light illumination. A photodetector composed of S-G/ZnS exhibits a photocurrent of 37 μA under 300 nm light illumination of 1.2 mW cm^−2^ in air at a bias of 1.0 V, which is higher 9.3 times than the photocurrent of a D-G/ZnS device, with stable on-off switching and excellent spectral selectivity. A photodetector based on MS-G/ZnS shows the high photocurrent of 0.115 mA at a bias of 1.0 V, which is greater by a factor of 10^7^ than the photocurrent of a graphene-free UV photodetector based on ZnS nanobelts operated at 20 V^16^. The photoexcited electrons in the conduction band of ZnS spontaneously undergo a charge-transfer process to graphene channels, which is the UV-selective photo-detection mechanism of our highly efficient device. Further studies on the development of UV photodetectors with high transparency and flexibilities are under way.

## Methods

### Graphene synthesis and transfer

Graphene was synthesized on a copper foil (Alpha Aecer, 99.999%) via a chemical vapor deposition (CVD) method at 1,000 °C using a mixed gas of CH_4_ (30 SCCM) and H_2_ (3 SCCM) as the reaction source. After coating a poly(methylmethacrylate) (PMMA) polymer layer on the as-grown graphene of one side of the Cu foil, the graphene on the other side of the Cu foil was removed by oxygen plasma. After the Cu foil was etched with an ammonium persulfate (APS) solution and rinsed with distilled water several times, the graphene was transferred on a target substrate. The sample was soaked in acetone to remove the PMMA layer.

### ZnS nanobelts synthesis

Single-crystalline wurtzite ZnS nanobelts were prepared via a hydrothermal process according to the reported method^41^ with some modification. A stock solution of Zn^2+^ was prepared by adding 1.0 mmol of ZnCl_2_(s) into 7.5 mL of water and 7.5 mL of C_2_H_4_(NH_2_)_2_(l) slowly with mild stirring; the stock solution was stirred further for 20 min to make a clear solution. A stock solution of S^2−^ was prepared by adding 1.0 mmol of S(s) into 15 mL of N_2_H_4_·H_2_O(l) with vigorous stirring for 20 min. The stock solutions of Zn^2+^ and S^2−^ were mixed together and stirred vigorously for 5 min. The mixture solution was then loaded into a Teflon-lined stainless-steel autoclave of 50 mL capacity, placed in a preheated oven at 180 °C for 9 h, and cooled to room temperature. A white precipitate produced in the reaction mixture was washed three times with water and two times with ethanol, vacuum-dried, and kept in a vial for further characterization.

### Device fabrication

Titanium (3 nm) and gold (30 nm) electrodes were deposited on a Si/SiO_2_ (300 nm) wafer by thermal evaporation, and graphene was transferred on the top of the electrodes. After spin coating of ZnS nanobelts dispersed ethanol on graphene for 20 s at 500 rpm, additional graphene was transferred on the top of the as-prepared sample.

### Characterization

Raman spectra and their mapping profiles were obtained by a Raman spectrometer (RM 1000-Invia, Renishaw, 514 nm), and AFM images were measured at a noncontact mode using an atomic force microscope (Park System, XE-100). TEM images and their SAED and FFT patterns were obtained with an electron microscope (JEOL, JEM- 3010) operating at 300 kV, and FE-SEM images were measured with another electron microscope (SUPRA, 55vp-zeiss). While optical microscopy images were obtained with a microscope (NIKON, Eclipse LV100ND), HRXRD patterns were obtained with a diffractometer (Bruker, D8 DISCOVER) using Cu Kα radiation (0.15418 nm). Extinction spectra were obtained with a UV-vis spectrophotometer (Scinco, S3100). The current-voltage behaviors of photodetectors were measured using a probe nanovoltmeter (Agilent, B2912A) with a Xe lamp (Schoeffel, LPS255HR) as the light source.

## Additional Information

**How to cite this article**: Kim, Y. *et al.* High-performance ultraviolet photodetectors based on solution-grown ZnS nanobelts sandwiched between graphene layers. *Sci. Rep.* **5**, 12345; doi: 10.1038/srep12345 (2015).

## Supplementary Material

Supplementary Information

## References

[b1] Mueller T., Xia F. & Avouris P. Graphene photodetectors for high-speed optical communications. Nat, Photon. 4, 297–301 (2010).

[b2] Wang J., Gudiksen M. S., Duan X., Cui Y. & Lieber C. M. Highly polarized photoluminescence and photodetection from single indium phosphide nanowires. Science 293, 1455–1457 (2001). 11520977 10.1126/science.1062340

[b3] Wang X., Tian W., Liao M., Bando Y. & Golberg D. Recent advances in solution-processed inorganic nanofilm photodetectors. Chem. Soc. Rev. 43, 1400–1422 (2014). 24356373 10.1039/c3cs60348b

[b4] Guo F. *et al.* A nanocomposite ultraviolet photodetector based on interfacial trap-controlled charge injection. Nature Nanotechnol. 7, 798–802 (2012). 23142945 10.1038/nnano.2012.187

[b5] Liang Y., Liang H., Xiao X. & Hark S. The epitaxial growth of ZnS nanowire arrays and their applications in UV-light detection. J. Mater. Chem. 22, 1199–1205 (2012).

[b6] Yang S., Gong J. & Deng Y. Opposite photocurrent response to ultraviolet and visible light. J. Mater. Chem. 22, 24522–24525 (2012).

[b7] Yang S., Gong J. & Deng Y. A sandwich-structured ultraviolet photodetector driven only by opposite heterojunctions. J. Mater. Chem. 22, 13899–13902 (2012).

[b8] Zhang Q. *et al.* Solution-processed graphene quantum dot deep-UV photodetectors. ACS Nano 9, 1561–1570 (2015). 25625624 10.1021/acsnano.5b00437

[b9] Konstantatos G. *et al.* Hybrid graphene-quantum dot phototransistors with ultrahigh gain. Nature Nanotechnol. 7, 363–368 (2012). 22562036 10.1038/nnano.2012.60

[b10] Yang S. *et al.* Environmentally stable/self-powered ultraviolet photodetectors with high sensitivity. Appl. Phys. Lett. 103, 143503 (2013).

[b11] Tian W. *et al.* Flexible ultraviolet photodetectors with broad photoresponse based on branched ZnS‐ZnO heterostructure nanofilms. Adv. Mater. 26, 3088–3093 (2014). 24523228 10.1002/adma.201305457

[b12] Hu L. *et al.* Stacking‐order‐dependent optoelectronic properties of bilayer nanofilm photodetectors made from hollow ZnS and ZnO microspheres. Adv. Mater. 24, 5872–5877 (2012). 22933411 10.1002/adma.201202749

[b13] Jie J. *et al.* Photoconductive characteristics of single-crystal CdS nanoribbons. Nano Lett. 6, 1887–1892 (2006). 16967996 10.1021/nl060867g

[b14] Soci C. *et al.* ZnO nanowire UV photodetectors with high internal gain. Nano Lett. 7, 1003–1009 (2007). 17358092 10.1021/nl070111x

[b15] Wang X. *et al.* Gas sensors, thermistor and photodetector based on ZnS nanowires. J. Mater. Chem. 22, 6845–6850 (2012).

[b16] Fang X. *et al.* Single‐crystalline ZnS nanobelts as ultraviolet‐light sensors. Adv. Mater. 21, 2034–2039 (2009).

[b17] Fang X. *et al.* An efficient way to assemble ZnS nanobelts as ultraviolet‐light sensors with enhanced photocurrent and stability. Adv. Funct. Mater. 20, 500–508 (2010).

[b18] Yu Y. *et al.* High-gain visible-blind UV photodetectors based on chlorine-doped n-type ZnS nanoribbons with tunable optoelectronic properties. J. Mater. Chem. 21, 12632–12638 (2011).

[b19] Lin Y.-Y. *et al.* Near-ultraviolet photodetector based on hybrid polymer/zinc oxide nanorods by low-temperature solution processes. Appl. Phys. Lett. 92, 233301 (2008).

[b20] Zhu H. *et al.* Metal−oxide−semiconductor-structured MgZnO ultraviolet photodetector with high internal gain. J. Phys. Chem. C 114, 7169–7172 (2010).

[b21] Li H.-G. *et al.* ZnO/poly (9, 9-dihexylfluorene) based inorganic/organic hybrid ultraviolet photodetector. Appl. Phys. Lett. 93, 153309 (2008).

[b22] Zhang F., Ding Y., Zhang Y., Zhang X. & Wang Z. L. Piezo-phototronic effect enhanced visible and ultraviolet photodetection using a ZnO–CdS core–shell micro/nanowire. ACS Nano 6, 9229–9236 (2012). 23020237 10.1021/nn3035765

[b23] Rigutti L. *et al.* Ultraviolet photodetector based on GaN/AlN quantum disks in a single nanowire. Nano Lett. 10, 2939–2943 (2010). 20617803 10.1021/nl1010977

[b24] Lee C., Wei X., Kysar J. W. & Hone J. Measurement of the elastic properties and intrinsic strength of monolayer graphene. Science 321, 385–388 (2008). 18635798 10.1126/science.1157996

[b25] Kim K. S. *et al.* Large-scale pattern growth of graphene films for stretchable transparent electrodes. Nature 457, 706–710 (2009). 19145232 10.1038/nature07719

[b26] Tan Y.-W. *et al.* Measurement of scattering rate and minimum conductivity in graphene. Phys. Rev. Lett. 99, 246803 (2007). 18233473 10.1103/PhysRevLett.99.246803

[b27] Morozov S. *et al.* Giant intrinsic carrier mobilities in graphene and its bilayer. Phys. Rev. Lett. 100, 016602 (2008). 18232798 10.1103/PhysRevLett.100.016602

[b28] Neto A. C., Guinea F., Peres N., Novoselov K. S. & Geim A. K. The electronic properties of graphene. Rev. Mod. Phys. 81, 109 (2009).

[b29] Adam S., Hwang E., Galitski V. & Sarma S. D. A self-consistent theory for graphene transport. Proc. Natl. Acad. Sci. USA. 104, 18392–18397 (2007). 18003926 10.1073/pnas.0704772104 PMC2141788

[b30] Nair R. *et al.* Fine structure constant defines visual transparency of graphene. Science 320, 1308–1308 (2008). 18388259 10.1126/science.1156965

[b31] Bunch J. S. *et al.* Impermeable atomic membranes from graphene sheets. Nano Lett. 8, 2458–2462 (2008). 18630972 10.1021/nl801457b

[b32] Chen S. *et al.* Oxidation resistance of graphene-coated Cu and Cu/Ni alloy. ACS Nano 5, 1321–1327 (2011). 21275384 10.1021/nn103028d

[b33] Kim S. J. *et al.* Simultaneous etching and doping by Cu-stabilizing agent for high-performance graphene-based transparent electrodes. Chem. Mater. 26, 2332–2336 (2014).

[b34] Son D. I., Yang H. Y., Kim T. W. & Park W. I. Photoresponse mechanisms of ultraviolet photodetectors based on colloidal ZnO quantum dot-graphene nanocomposites. Appl. Phys. Lett. 102, 021105 (2013).

[b35] Babichev A. *et al.* GaN nanowire ultraviolet photodetector with a graphene transparent contact. Appl. Phys. Lett. 103, 201103 (2013).

[b36] Zhan Z., Zheng L., Pan Y., Sun G. & Li L. Self-powered, visible-light photodetector based on thermally reduced graphene oxide–ZnO (rGO–ZnO) hybrid nanostructure. J. Mater. Chem. 22, 2589–2595 (2012).

[b37] Ferrari A. *et al.* Raman spectrum of graphene and graphene layers. Phys. Rev. Lett. 97, 187401 (2006). 17155573 10.1103/PhysRevLett.97.187401

[b38] Xiong Q., Wang J., Reese O., Lew Yan Voon L. & Eklund P. Raman scattering from surface phonons in rectangular cross-sectional w-ZnS nanowires. Nano Lett. 4, 1991–1996 (2004).

[b39] Li L. *et al.* Electrical transport and high-performance photoconductivity in individual ZrS_2_ nanobelts. Adv. Mater. 22, 4151–4156 (2010). 20730817 10.1002/adma.201001413

[b40] Geim A. K. & Novoselov K. S. The rise of graphene. Nat. Mater. 6, 183–191 (2007). 17330084 10.1038/nmat1849

[b41] Kim Y. & Jang D.-J. Facile one-step hydrothermal fabrication of single-crystalline ZnS nanobelts with narrow band-edge luminescence. RSC Adv. 3, 16945–16948 (2013).

