# Supplementary material for: High-performance ultraviolet photodetectors based on solution-grown ZnS nanobelts sandwiched between graphene layers
Source: Sci Rep. 2015 Jul 22;5:12345. doi: 10.1038/srep12345 (PMC4510574; doi:10.1038/srep12345)
Supplement: Supplementary Information [file srep12345-s1.pdf]

Supporting Information

High-performance ultraviolet photodetectors based on solution-grown ZnS nanobelts sandwiched between graphene layers

Yeonho Kim[1,†], Sang Jin Kim[1,†], Sung-Pyo Cho[2], Byung Hee Hong[1,] & Du-Jeon Jang[1,]**

[1]Department of Chemistry and [2]National Center for Inter-University Research Facilities, Seoul National University, Seoul 151-747, Korea.

[†]These authors contributed equally to this work.

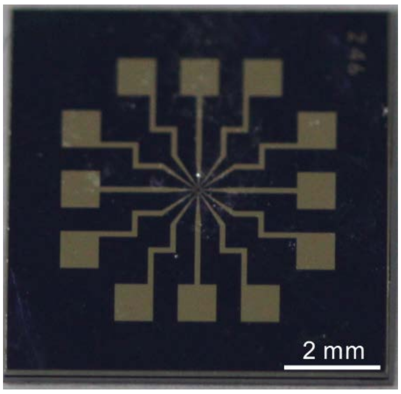

Supplementary Figure 1 | Digital image of an as-fabricated device with a channel length of 80 μm.

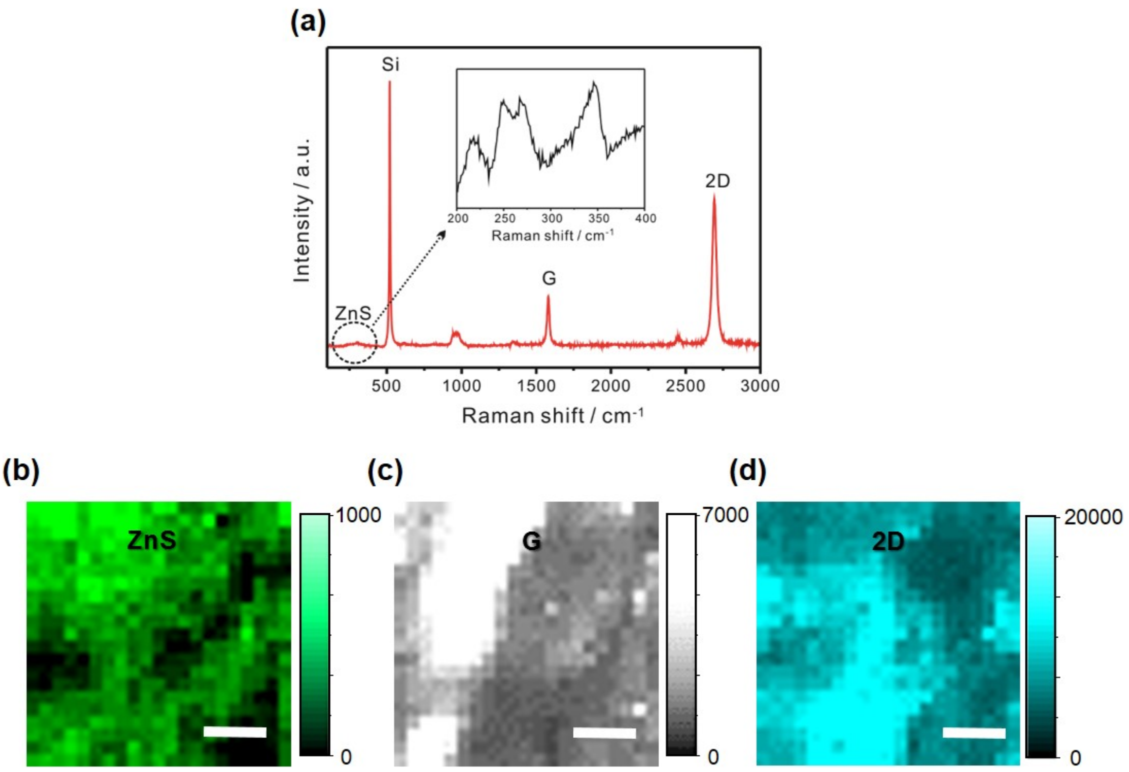

Supplementary Figure 2 | (a) Raman spectrum of a G/ZnS sandwich-structured device and mapping profiles (scale bar = 5 μm) of (b) the ZnS, (c) the G band, and (d) the 2D band.

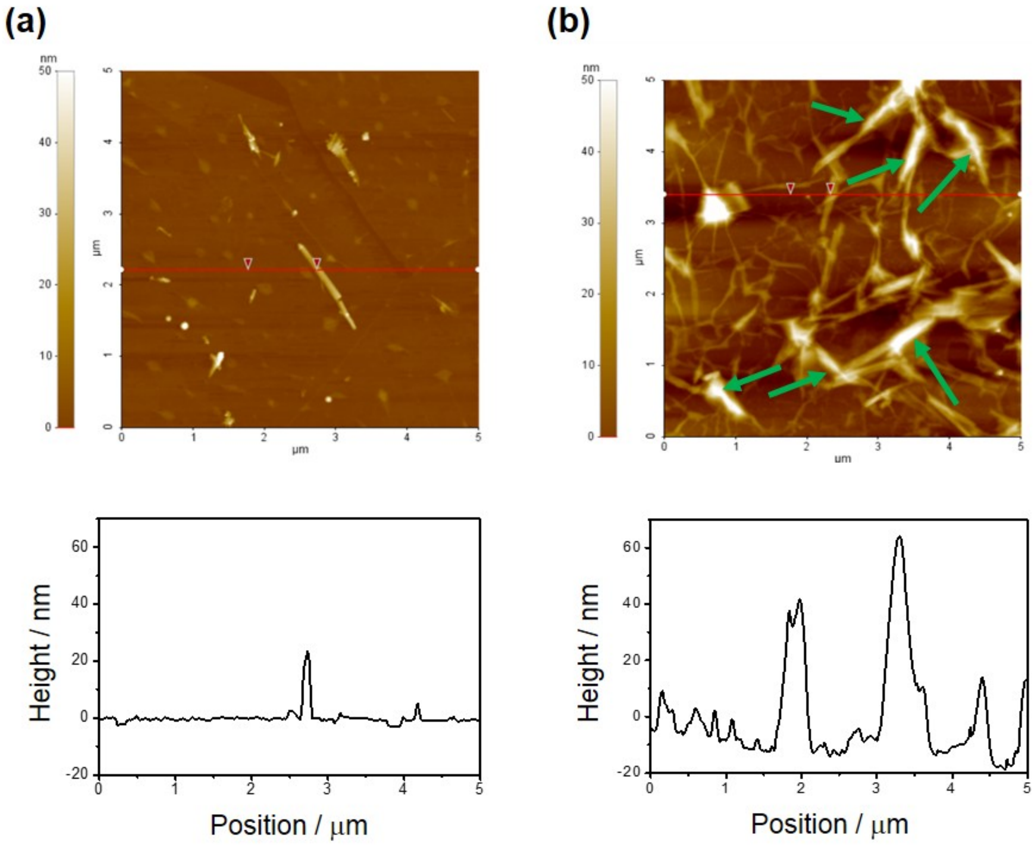

Supplementary Figure 3 | AFM images (top) and their corresponding height profiles (bottom) of (a) D-G/ZnS and (b) composite S-G/ZnS. The height profiles were scanned along the red lines, and the green arrows indicate tent-like structures of graphene.

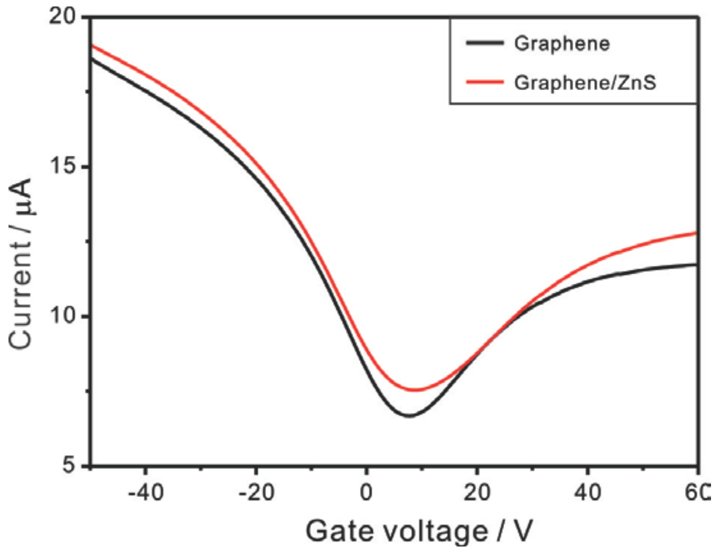

Supplementary Figure 4 | Current-voltage characteristic curves of (black) graphene and (red) composite G/ZnS.

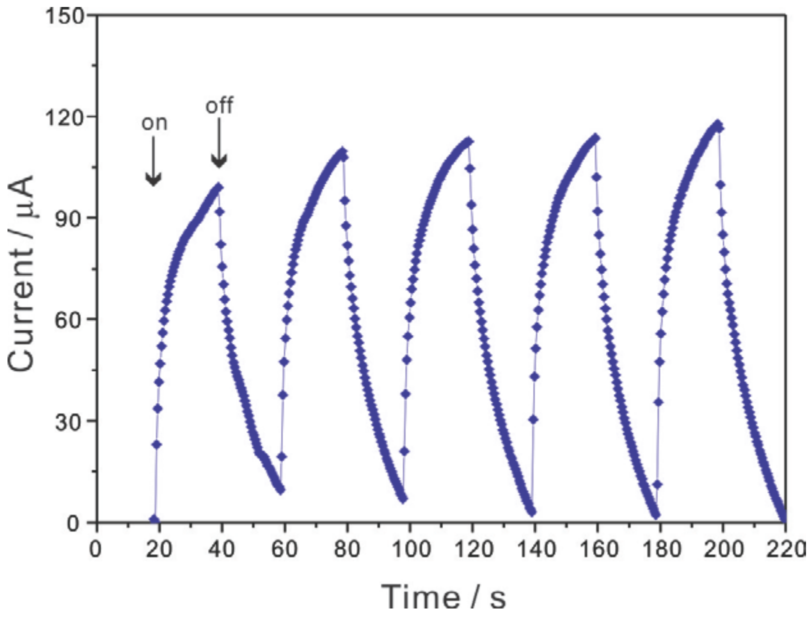

Supplementary Figure 5 | Response behavior of a MS-G/ZnS photodetector measured in air at a bias of 1.0 V under 300 nm light illumination of 1.2 mW cm^{-2}.

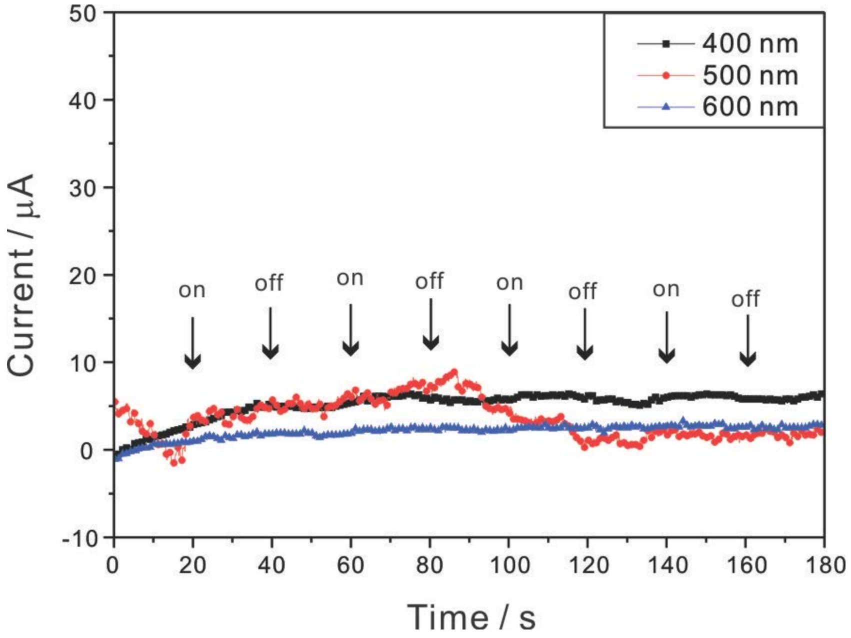

Supplementary Figure 6 | Response behaviors of a S-G/ZnS photodetector measured in air at a bias of 1.0 V under 400 (black), 500 (red), and 600 nm (blue) light illumination of 1.2 mW cm^{-2}.

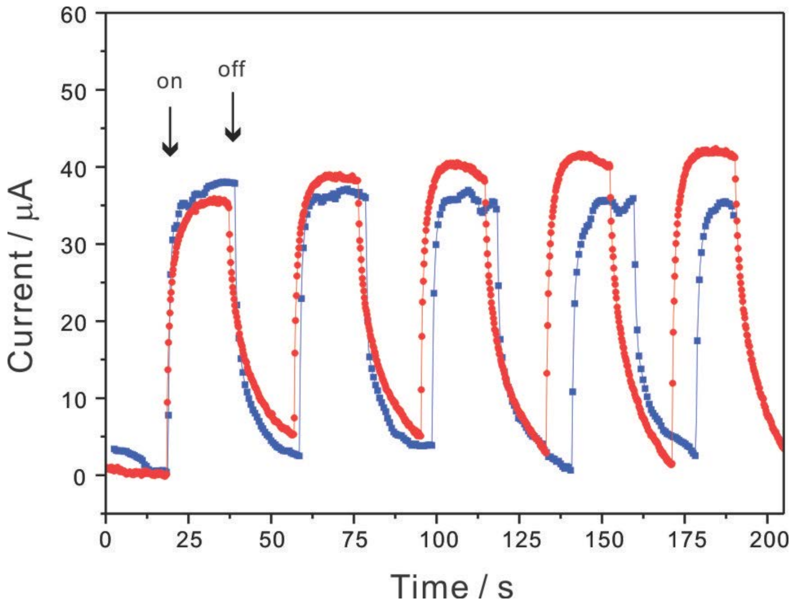

Supplementary Figure 7 | Response behaviors of as-prepared (blue) and six months-old (red) S-G/ZnS photodetectors measured in air at a bias of 1.0 V under 300 nm light illumination of 1.2 mW cm^{-2}.
